# Efficacy of a Single-Loading Dose of Magnesium Sulphate in Preventing Convulsions in Women With Eclampsia: A Quasi-experimental Study

**DOI:** 10.7759/cureus.95460

**Published:** 2025-10-26

**Authors:** Naila Khan, Wajeeha Yahya, Rabia Hammad, Rifaat Rehan, Asma Iqbal, Salma Naseem, Amna Abdullah

**Affiliations:** 1 Department of Obstetrics and Gynaecology, Dr. Ruth Katherina Martha (KM) Pfau, Civil Hospital Karachi, Karachi, PAK; 2 Department of Obstetrics and Gynaecology, Aga Khan University Hospitals, Karachi, PAK; 3 Department of Obstetrics and Gynaecology, Karachi Medical and Dental College, Abbasi Shaheed Hospital, Karachi, PAK; 4 Department of Obstetrics and Gynaecology, Aga Khan Hospital for Women and Children, Kharadar, Karachi, PAK; 5 Department of Obstetrics and Gynaecology, Kharadar General Hospital, Karachi, PAK

**Keywords:** anticonvulsant therapy, convulsions, eclampsia, magnesium sulphate, resource-limited settings

## Abstract

Eclampsia remains a major contributor to maternal morbidity and mortality in low-resource settings. Magnesium sulphate is the anticonvulsant of choice; however, prolonged maintenance regimens can be challenging to implement. This study aimed to evaluate the efficacy of a single-loading dose of magnesium sulphate in preventing recurrent convulsions among women presenting with eclampsia. This analytical quasi-experimental study was conducted in the Obstetrics and Gynecology units of Civil Hospital, Karachi, from September 8, 2021, to March 7, 2022. A total of 113 women aged 18-40 years, with singleton pregnancies between 37 and 41 weeks of gestation and diagnosed with eclampsia, were enrolled through non-probability consecutive sampling. Each participant received a single IV dose of magnesium sulphate (4 g IV over 8-10 minutes followed by 4 g intramuscular (IM)). No maintenance dose was given. Efficacy, defined as the absence of recurrent convulsions within 24 hours, was recorded. Data were analyzed using SPSS version 26.0, with chi-square tests applied to assess associations (*p* ≤ 0.05 considered significant). The mean age of participants was 28.93 ± 4.46 years, and the mean gestational age was 38.31 ± 1.20 weeks. Most women were multiparous (87.6% with parity 0-2), booked (54.0%), and from rural areas (61.1%). Overall, efficacy was observed in 91 women (80.5%). Most variables, including age, gestational age, parity, BMI, history of eclampsia, place of residence, and education level, were not significantly associated with efficacy (*p* > 0.05). However, booking status (unbooked: 88.3% vs. booked: 79.2%; *p* = 0.018) and monthly income (<20,000 Pakistani rupee (PKR): 84.8% vs. >40,000 PKR: 87.5%; *p* = 0.041) showed statistically significant associations with treatment outcomes. A single-loading dose of magnesium sulphate was effective in preventing recurrent convulsions in the majority of women with eclampsia. Given its simplicity and high efficacy, this regimen may be particularly useful in resource-limited settings where maintenance therapy is difficult to administer. Larger multicenter studies are recommended to validate these findings and assess long-term outcomes.

## Introduction

Hypertensive disorders of pregnancy remain a major concern among obstetricians and are a leading cause of maternal and perinatal morbidity and mortality worldwide. They account for approximately 10-25% of maternal deaths in low- and middle-income countries, with eclamptic seizures being a major cause [1]. In developed countries, where modern antenatal care allows for early detection and treatment of preeclampsia, eclampsia has become rare (1 in 2000 deliveries). In contrast, in many developing countries, the incidence ranges between 1 in 100 and 1 in 1700 deliveries, particularly in rural areas where women may present in deep coma after multiple seizures at home [2].

The cornerstone of eclampsia management is seizure control. In recent years, several large studies have demonstrated the superiority of magnesium sulphate over other anticonvulsants, establishing it as the first-line drug of choice for preventing and treating eclamptic seizures [3]. Among the two internationally recommended regimens, Pritchard’s and Zuspan’s, the former is more widely used in resource-poor settings [4]. However, the conventional “high-dose” Pritchard regimen, standardized for Western women, has been associated with dose-related toxicities such as respiratory, renal, and neuromuscular complications when applied to lean, malnourished women in low- and middle-income countries [5]. This has led to reduced acceptance and the exploration of simplified dosing strategies. Recent studies have suggested that a single-loading dose of magnesium sulphate may be sufficient to prevent further convulsions [6,7].

Evidence from international literature shows efficacy rates of 74-88% for a single-loading dose in preventing recurrent convulsions [8-10]. Magnesium sulphate is the most commonly used anticonvulsant in our setting, yet existing evidence is limited by differences in population characteristics, health system capacity, and accessibility to care. Previous local data on this approach are more than five years old and may not reflect current trends. Given the high burden of eclampsia in rural and low-socioeconomic populations in our region, there is a clear need to reassess the role of a simplified magnesium sulphate regimen. Therefore, the objective of this study was to determine the efficacy of a single-loading dose of magnesium sulphate in preventing recurrent convulsions in women with eclampsia in our local population.

## Materials and methods

Study design and setting

This analytical quasi-experimental (single-arm prospective) study was conducted in the Obstetrics and Gynecology units of Civil Hospital, Karachi, from 8 September 2021 to 7 March 2022. A sample size of 113 was calculated using the WHO sample size calculator for a single proportion, with a 95% confidence level, 6% margin of error, and an expected efficacy of a single-loading dose of magnesium sulphate in preventing convulsions among eclamptic women of 80%. A non-probability consecutive sampling technique was employed. Ethical approval was obtained from the Research Evaluation Unit, CPSP, under reference number CPSP/REU/OBG-2020-183-9877. Written informed consent was obtained from all participants.

Eligibility criteria

Inclusion criteria comprised women aged 18-40 years with singleton pregnancies between 37 and 41 weeks of gestation (based on the last menstrual period and confirmed by ultrasound), parity 0-5, both booked and unbooked, and presenting with eclampsia as per the operational definition.

Exclusion criteria included prior receipt of magnesium sulphate or other anticonvulsants before admission; contraindications to magnesium sulphate (respiratory depression, renal failure, heart block, severe hepatitis, hypersensitivity); history of chronic hypertension; and convulsions due to other causes such as epilepsy, meningitis, or intracranial space-occupying lesions.

Data collection

After approval from the Institutional Ethical Review Committee, 113 eligible women were enrolled after obtaining informed consent from their attendants. Each participant received a single-loading dose of magnesium sulphate consisting of 4 g (20 ml of 20%) administered intravenously over 8-10 minutes, followed by 4 g of 50% solution intramuscularly, making a total loading dose of 8 g. No maintenance dose was given. Treatment efficacy was assessed after 24 hours according to the operational definition. Women who developed recurrent convulsions after the initial dose were managed according to hospital protocol, which included an additional 2 g intravenous dose of magnesium sulphate. If seizures persisted, diazepam (5-10 mg IV) was administered as rescue therapy. No other antiepileptic drugs were required.

Sociodemographic and clinical data, including age, gestational age, gravida, parity, booking status, place of residence (rural/urban), BMI, previous history of eclampsia, monthly income (<20,000 / 20,000-40,000 / >40,000 PKR), education level (illiterate / primary / middle / matric and above), and treatment efficacy (yes/no), were recorded on a pre-designed proforma.

Statistical analysis

Data were analyzed using SPSS version 25.0. Continuous variables (age, parity, gestational age, height, weight, BMI) were summarized as mean ± SD. Categorical variables (place of residence, booking status, income, education level, history of eclampsia, and efficacy) were presented as frequencies and percentages.

Stratification was performed for age, gestational age, parity, BMI, history of eclampsia, residence, booking status, income, and education level to assess potential effect modifiers. The Chi-square test was applied, and a p-value ≤ 0.05 was considered statistically significant.

## Results

A total of 113 women with eclampsia were enrolled. The mean age was 28.93 ± 4.46 years, and the mean gestational age was 38.31 ± 1.20 weeks. The majority presented at 37-39 weeks of gestation (78.8%), had a parity of 0-2 (87.6%), and a BMI >30 kg/m² (54.0%). A past history of eclampsia was present in 37.2% of patients. resided in rural areas (61.1%). With respect to socioeconomic indicators, 60.2% had a monthly family income <20,000 PKR, and 19.5% were illiterate.

Treatment efficacy did not differ significantly by age group, though 77.8% of women aged 18-30 years responded compared to 91.3% of those aged 31-40 years (χ² = 2.14, p = 0.144). Similarly, gestational age was not associated with outcome; efficacy was 83.1% at 37-39 weeks and 70.8% at 40-41 weeks (χ² = 1.83, p = 0.176). Parity showed no significant association, with efficacy of 81.8% among women with parity 0-2 and 71.4% among those with parity 3-5 (χ² = 0.84, p = 0.358). Likewise, BMI was not related to efficacy: 76.9% in BMI ≤30 versus 83.6% in BMI >30 (χ² = 0.80, p = 0.371). A history of eclampsia also did not influence outcomes, with efficacy rates of 78.6% among women with a prior history and 81.7% among those without (χ² = 0.16, p = 0.686).

By contrast, booking status was significantly associated with efficacy. Unbooked women had higher response rates (88.3%) compared to booked women (79.2%; χ² = 5.60, p = 0.018). Socioeconomic status was also significantly associated with outcomes, with efficacy of 84.8% among women earning <20,000 PKR/month, 81.6% in the 20,000-40,000 PKR group, and 87.5% in those earning >40,000 PKR (χ² = 6.35, p = 0.041). No association was observed with place of residence (81.2% rural vs. 79.5% urban; χ² = 0.04, p = 0.833) or level of education (90.9% illiterate, 78.6% primary, 79.1% middle, 76.5% matric & above; χ² = 1.96, p = 0.580) (Table 1).

**Table 1:** Association of baseline characteristics with treatment efficacy following a single-loading dose of magnesium sulphate (analyzed using the Chi-square test). Note: *p-value < 0.05 was considered statistically significant.

Variable	Efficacy Yes (n = 91)	Efficacy No (n = 22)	p-value
Age (years)			
18-30	70 (77.8%)	20 (22.2%)	0.144
31-40	21 (91.3%)	2 (8.7%)	
Gestational age (weeks)			
37-39	74 (83.1%)	15 (16.9%)	0.176
40-41	17 (70.8%)	7 (29.2%)	
Parity			
0-2	81 (81.8%)	18 (18.2%)	0.358
3-5	10 (71.4%)	4 (28.6%)	
BMI (kg/m²)			
≤30	40 (76.9%)	12 (23.1%)	0.371
>30	51 (83.6%)	10 (16.4%)	
History of eclampsia			
Yes	33 (78.6%)	9 (21.4%)	0.686
No	58 (81.7%)	13 (18.3%)	
Booking status			
Unbooked	53 (88.3%)	7 (11.7%)	0.018*
Booked	42 (79.2%)	11 (20.8%)	
Place of residence			
Rural	56 (81.2%)	13 (18.8%)	0.833
Urban	35 (79.5%)	9 (20.5%)	
Monthly income (PKR)			
<20,000	50 (84.8%)	9 (15.2%)	0.041*
20,000-40,000	31 (81.6%)	7 (18.4%)	
>40,000	14 (87.5%)	2 (12.5%)	
Education level			
Illiterate	20 (90.9%)	2 (9.1%)	0.58
Primary	11 (78.6%)	3 (21.4%)	
Middle	34 (79.1%)	9 (20.9%)	
Matric and above	26 (80.5%)	8 (23.5%)	

Among the 113 women included in the study, 111 (98.2%) resulted in live births, while 2 (1.8%) experienced stillbirths, consistent with the hospital’s baseline rate of approximately 2% among hypertensive pregnancies. No cases of neonatal death were observed during the immediate postnatal period. All mothers who responded to the single-loading dose of magnesium sulphate remained seizure-free within 24 hours. Among the 22 women who experienced recurrent convulsions, all received an additional 2 g/hour IV dose of magnesium sulphate; 3 (2.7%) also required diazepam (5-10 mg IV) as rescue therapy. No other antiepileptic drugs were used. Maternal recovery was uneventful in most cases, with no reported mortality or severe magnesium-related toxicity.

## Discussion

Current strategies for the prevention of pre-eclampsia can be broadly classified into antenatal surveillance, lifestyle modification, nutritional supplementation, and pharmacological therapy. The only definitive treatment for pre-eclampsia or eclampsia is termination of pregnancy. The aim of interventions in eclampsia is to prevent further seizures, minimize complications, and optimize the timing of delivery for the baby. Standard care for severe pre-eclampsia/eclampsia includes an anticonvulsant drug to control immediate fits, followed by maintenance treatment to prevent recurrence [11]. Magnesium sulphate has become the first-line anticonvulsant for the treatment of severe pre-eclampsia and eclampsia, while diazepam remains an alternative when magnesium sulphate is unavailable [12].

As most of our population presents with eclampsia due to lack of awareness and inadequate antenatal care, and because we frequently encounter such cases in our setting, we conducted this study to determine the efficacy of a single-loading dose of magnesium sulphate in preventing recurrent convulsions. The age range in our study was 18-40 years, with a mean age of 28.93 ± 4.46 years. These results were slightly higher than those reported by Imaralu JO et al. [10], who found a mean age of 24 years. Khan I et al. [13], in his study, reported a mean age of 23 years, which is considerably lower than that observed in our study. Sharma et al. [14] found a similar mean age of 28 years, consistent with our findings.

The mean gestational age was 38.3 ± 1.2 weeks, which is consistent with Khan I et al. [13], who also reported 38 weeks. By contrast, Duley L et al. [15] observed a mean gestational age of 33 weeks, reflecting earlier onset in some populations. Traditionally, the most common presentation of eclampsia has been at 28-32 weeks, but our findings suggest a shift toward term gestations in our population. This higher mean gestational age may reflect delayed presentation to tertiary care centers, as many women in our population seek obstetric attention only after the onset of convulsions or labor. Additionally, regional differences in referral patterns and access to emergency obstetric care can influence the timing of admission for eclamptic patients. Similar observations of term-gestation eclampsia have been reported in South Asian cohorts, suggesting a contextual shift related to evolving maternal health-care utilization patterns [16,17].

We observed that most cases occurred in women from rural areas (61.1%) and among those of low socioeconomic status (60.2% with monthly income <20,000 PKR). These findings are consistent with Sharma R et al. [14], who also identified rural residence and low income as risk factors, likely due to limited healthcare access and poor awareness of antenatal services.

In our study, the efficacy of a single-loading dose of magnesium sulphate was observed in 91 (80.5%) women. This aligns with the findings of a meta-analysis by Duley L et al. [15], which confirmed a substantial reduction in the risk of recurrent seizures (RR 0.44, 95% CI 0.34-0.57) with magnesium sulphate. According to Getaneh W et al. [18], none of the women with severe pre-eclampsia convulsed after initiation of magnesium sulphate, while four eclamptic women experienced recurrence. Among the 22 women (19.5%) who experienced recurrent convulsions after the single-loading dose, an additional 2 g/hour IV dose of magnesium sulphate was administered, followed by diazepam in refractory cases. Most responded to this supplemental therapy without further complications. Regarding fetomaternal outcomes, all mothers recovered without mortality or major morbidity. The overall stillbirth rate in the study population was 2%, consistent with the background rate of hypertensive pregnancies in our institution. Neonatal outcomes were generally favorable, with the majority of infants having satisfactory Apgar scores at five minutes and no significant differences between the efficacy and non-efficacy groups. These findings suggest that while a single-loading dose was effective for most women, close monitoring and readiness for additional anticonvulsant therapy remain essential for optimal maternal and fetal outcomes. Similar observations have been reported by Begum MR et al. [19] and Shoaib T et al. [7], who found low recurrence rates and comparable perinatal outcomes with modified magnesium sulphate regimens in resource-limited settings.

The findings of this study should be interpreted in light of some limitations. First, the sample size was modest, and the number of women experiencing recurrent convulsions was small, limiting statistical power. Second, as a single-center study, the results may not be generalizable to other populations or healthcare systems. Third, we assessed short-term efficacy (24 hours), but long-term maternal and neonatal outcomes could not be evaluated. Furthermore, some potential predictors had small cell counts, limiting robust subgroup analyses. In addition, serum magnesium levels were not measured due to logistic and cost constraints, which limited assessment of pharmacologic correlation with clinical efficacy. Future comparative studies evaluating the simplified 8 g single-dose protocol against the standard 12 g Pritchard regimen, including serum level monitoring, would provide more comprehensive evidence regarding optimal dosing and safety. Despite these limitations, our findings highlight that a single-loading dose of magnesium sulphate prevented recurrent seizures in 80.5% of women, which is comparable to international data.

## Conclusions

In this study, a single-loading dose of magnesium sulphate demonstrated high efficacy in preventing recurrent convulsions among women with eclampsia. Treatment response was particularly favorable in younger women, those with lower socioeconomic status, and rural populations, groups that are often underserved in terms of healthcare access. These findings suggest that a simplified MgSO₄ regimen could be a feasible strategy in resource-limited settings where prolonged maintenance therapy is challenging. However, the study’s modest sample size, single-center design, and short follow-up period limit its generalizability. Larger multicenter trials are needed to confirm these results and to evaluate maternal and perinatal outcomes beyond the immediate postpartum period.
